# Alpha-Gal on the Rise: The Alarming Growth of Alpha-Gal Syndrome in High-Risk Regions

**DOI:** 10.7759/cureus.88415

**Published:** 2025-07-21

**Authors:** Myles Ross, Gianna Daley, Madison L Burnard, Ayushi Sen, Elizabeth Beyene, Mekdem Bisrat, Samrawit W Zinabu, Mahlet Abrie, Miriam Michael

**Affiliations:** 1 Internal Medicine, Howard University College of Medicine, Washington, DC, USA; 2 School of Medicine, Howard University College of Medicine, Washington, DC, USA; 3 Internal Medicine, American University of Antigua College of Medicine, St. John's, ATG; 4 Internal Medicine, Howard University Hospital, Washington, DC, USA; 5 Ophthalmology, Eka Kotebe General Hospital, Addis Ababa, ETH

**Keywords:** alpha-gal, delayed hypersensitivity, ige, mammalian meat allergy, tick

## Abstract

Background

Mammalian meat allergy (MMA), increasingly linked to delayed hypersensitivity reactions such as alpha-gal syndrome, is a growing public health concern. This study evaluates the incidence, prevalence, and incidence rate of MMA in a large, diverse population of over 114 million individuals across two time periods (2015-2020 and 2021-2025), with stratification by age, sex, race, and ethnicity.

Methods

Using longitudinal electronic health record data, we calculated incidence proportion, prevalence, and incidence rate (cases per person-day) for MMA diagnoses. Analyses were stratified by demographic factors and compared across time windows. Predictive modeling was used to estimate future trends in high-risk populations.

Results

MMA increased dramatically across all demographics. Overall incidence proportion rose by over 5,500%, with the most substantial increases observed in individuals over 40 years old. Age-stratified analysis within racial groups revealed particularly sharp increases among Black (9,530%) and Hispanic (7,678%) adults over 40. Females experienced a steeper rise in incidence rate (11,169%) than males (7,426%). Predictive modeling projects continued growth through 2030, with incidence proportions in Black and Hispanic populations expected to increase by 57% and 72%, respectively.

Conclusion

MMA is emerging as a rapidly increasing diagnosis, particularly among middle-aged and older adults in racially diverse populations. These findings highlight the need for targeted public health efforts, including clinician education, adult-focused screening, and prevention strategies - especially in communities with rising allergy rates. Enhanced awareness and early identification are critical to mitigating the rising burden of this potentially life-threatening allergic condition.

## Introduction

Alpha-gal syndrome (AGS) is an allergy characterized by hypersensitivity to mammalian meat. Patients suffering from the syndrome develop IgE antibodies against galactose-α-1,3-galactose (alpha-gal), a carbohydrate found in non-primate mammal cells [1]. The mechanism behind the development of the allergy is thought to be mediated by tick bites. In America, specifically, the lone star tick has been identified as a significant agent [2]. Sensitization is believed to involve repeated exposures that introduce alpha-gal-containing proteins and trigger IgE-mediated immune responses [3].

Clinically, AGS typically presents as a delayed hypersensitivity reaction occurring three to six hours after ingestion of mammalian meat, manifesting with urticaria, angioedema, gastrointestinal distress, and, in severe cases, anaphylaxis - features that often complicate timely recognition and diagnosis [1,2].

Between 2010 and 2018, there were more than 34,000 suspected cases of AGS in the United States [4,5]. Of these cases, men were more likely to have a positive test result [5]. Along with further testing between 2017 and 2021, estimates by the Centers for Disease Control and Prevention suggest that between 2010 and 2022, there were over 110,000 suspected cases of AGS in the United States; however, this number may exceed 450,000 due to underdiagnosis and lack of information [4]. Between 2017 and 2022, 295,400 individuals in the United States were tested, with 42% of men and 24% of women receiving a positive result, indicating a higher prevalence among males, despite a greater number of females undergoing testing [4]. Notably, the likelihood of a positive test increased with age, with nearly 45% of individuals aged 70 and older testing positive, compared to a mean age of 48 years for those with a positive result [4].

Racial disparities in AGS IgE sensitization were evident in a national study of 3,000 U.S. military recruits, where White participants had a significantly higher sensitization rate (7.5%) compared to Black (2.6%), Hispanic (2.4%), and Asian/Pacific Islander (4.4%) participants [6]. Multivariable logistic regression confirmed that Black and Hispanic races were independently associated with a lower likelihood of sensitization compared to White race, even when controlled for age, sex, geographic region, and rural versus urban residence [6]. These findings suggest that sociodemographic factors and biological differences may contribute to observed disparities, though further research is needed.

The lone star tick has been linked to other disease entities in the past, including, but not limited to, *Francisella tularensis*, southern tick-associated rash illness, and, more recently, Rocky Mountain spotted fever [7]. Traditionally found primarily in southern states, lone star ticks have been seen more frequently in northeastern states such as Delaware and Connecticut [8,9]. This shift may be due to rising global temperatures, which contribute to more favorable conditions for tick survival and growth [10]. The warming pattern may also increase the duration of tick activity, further extending the period of disease transmission. In addition, lone star ticks can lay thousands of eggs at a time, which facilitates their spread. A study of lone star ticks in New York identified genetic differences that may suggest adaptation conferring resistance to the northern climate [11].

Given the limited large-scale studies on the evolving trends and demographic patterns of AGS, this study aimed to evaluate the incidence, prevalence, and demographic distribution of mammalian meat allergy (MMA) in a national cohort of over 114 million patients. By stratifying trends by age, sex, race, and ethnicity, we sought to identify high-risk groups and provide insights to guide targeted public health efforts and clinical interventions.

## Materials and methods

This retrospective cohort study utilized de-identified electronic health records (EHRs) from the TriNetX Global Collaborative Network, which includes data from 124 healthcare organizations (HCOs), representing a total of 114,696,176 patients. Patients were eligible for inclusion if they had at least one documented healthcare encounter between January 1, 2015, and February 28, 2025, identified using the TriNetX-defined concept “Visit (TNX: Visit).” Patients with a prior diagnosis of MMA, identified through a lookback period before each observation window, were excluded to ensure capture of incident cases and to avoid misclassification of prevalent cases as new diagnoses.

To evaluate trends over time, two discrete observation periods were defined: January 1, 2015, to December 31, 2020, and January 1, 2021, to February 28, 2025. A lookback period was applied from any time prior to the start of each observation window, through one day before its beginning, allowing identification of newly diagnosed cases.

The primary outcome was a diagnosis of MMA, defined using the ICD-10 code Z91.014 (allergy to mammalian meats). No additional exclusion codes were applied. We acknowledge that reliance on a single diagnostic code may lead to under- or overestimation, due to potential miscoding or overdiagnosis. Cohorts were stratified by age, sex, race, and ethnicity.

Outcome measures included incidence proportion (new cases per total at-risk population), incidence rate (new cases per person-time), and period prevalence (total cases, including both new and existing, within the observation window). Analyses were conducted using TriNetX’s Incidence and Prevalence Analysis tool. TriNetX employs patient-level deduplication, using unique identifiers, to ensure that repeat visits or multiple encounters by the same individual are not counted as separate cases.

Additionally, future trends for the 2025-2030 period were estimated using TriNetX’s automated, machine learning-based forecasting tool, which primarily applies exponential smoothing and ARIMA (AutoRegressive Integrated Moving Average) time-series methods, derived from historical incidence data, to generate projections. Because this forecasting tool internally selects optimal parameters and performs cross-validation within a proprietary system, specific model settings were not externally accessible.

Because this study involved only the secondary analysis of de-identified data and did not include direct interaction with human subjects, Institutional Review Board (IRB) approval and informed consent were not required. This determination is consistent with the U.S. HIPAA Privacy Rule’s standard for de-identification and the use of data for research purposes. Formal statistical tests of significance between groups (e.g., incidence rate ratios or survival analyses) were not performed, as this study aimed to characterize descriptive epidemiologic trends. Future research should incorporate these methods to quantify differences and potential interactions among demographic groups.

## Results

Between 2015 and 2025, the incidence and prevalence of MMA increased significantly across all demographics. Based on TriNetX analysis of a national cohort of 114,696,176 patients, the number of new MMA diagnoses rose from 180 cases during 2015-2020 to 10,132 cases during 2021-2025. This corresponds to a 5,520% increase in incidence proportion and a 5,566% increase in prevalence, reflecting substantial growth in both new diagnoses and the total number of individuals living with the condition. The incidence rate (cases per person-day) increased by 8,950%, indicating that new diagnoses became increasingly frequent relative to time and population exposure.

**Figure 1:**
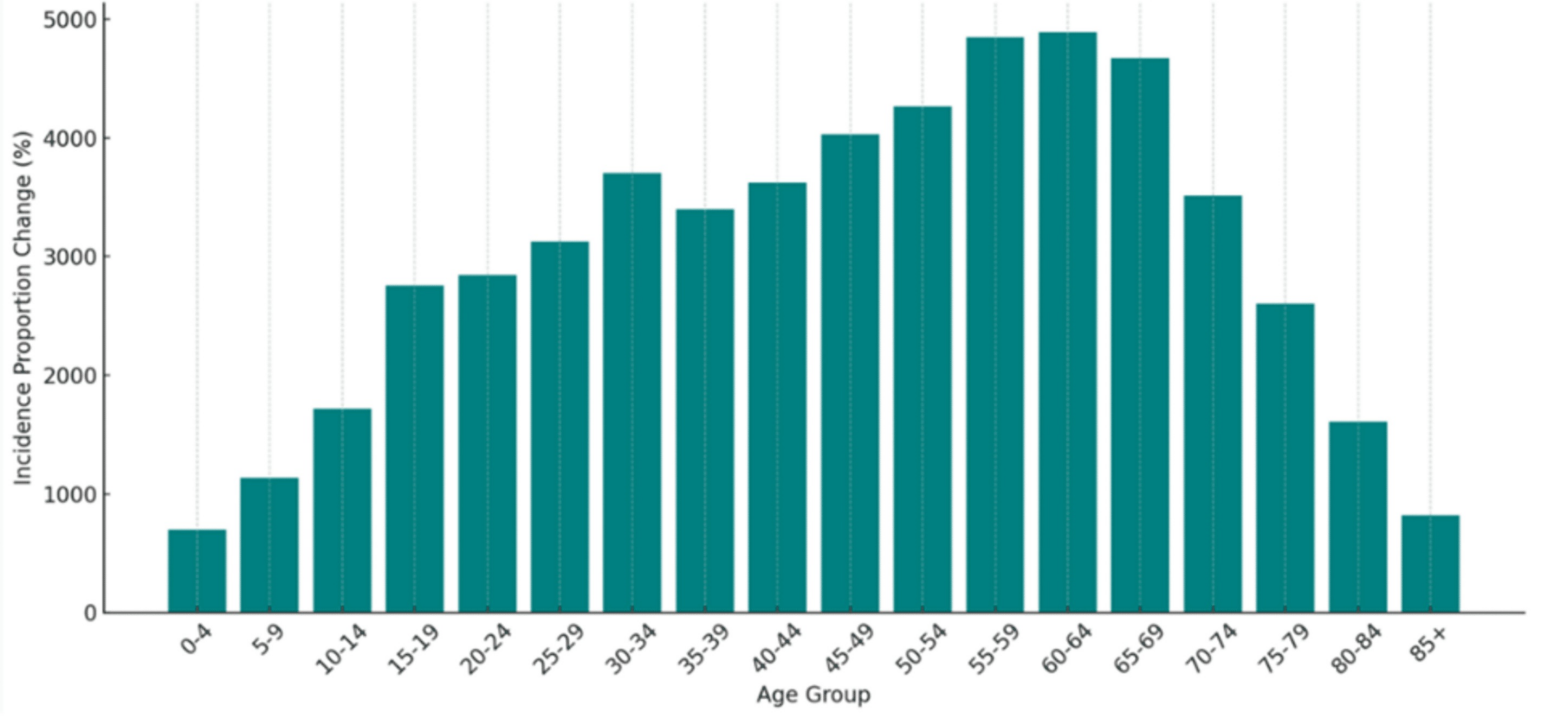
Change in Incidence of Mammalian Meat Allergy by Age Group

**Figure 2:**
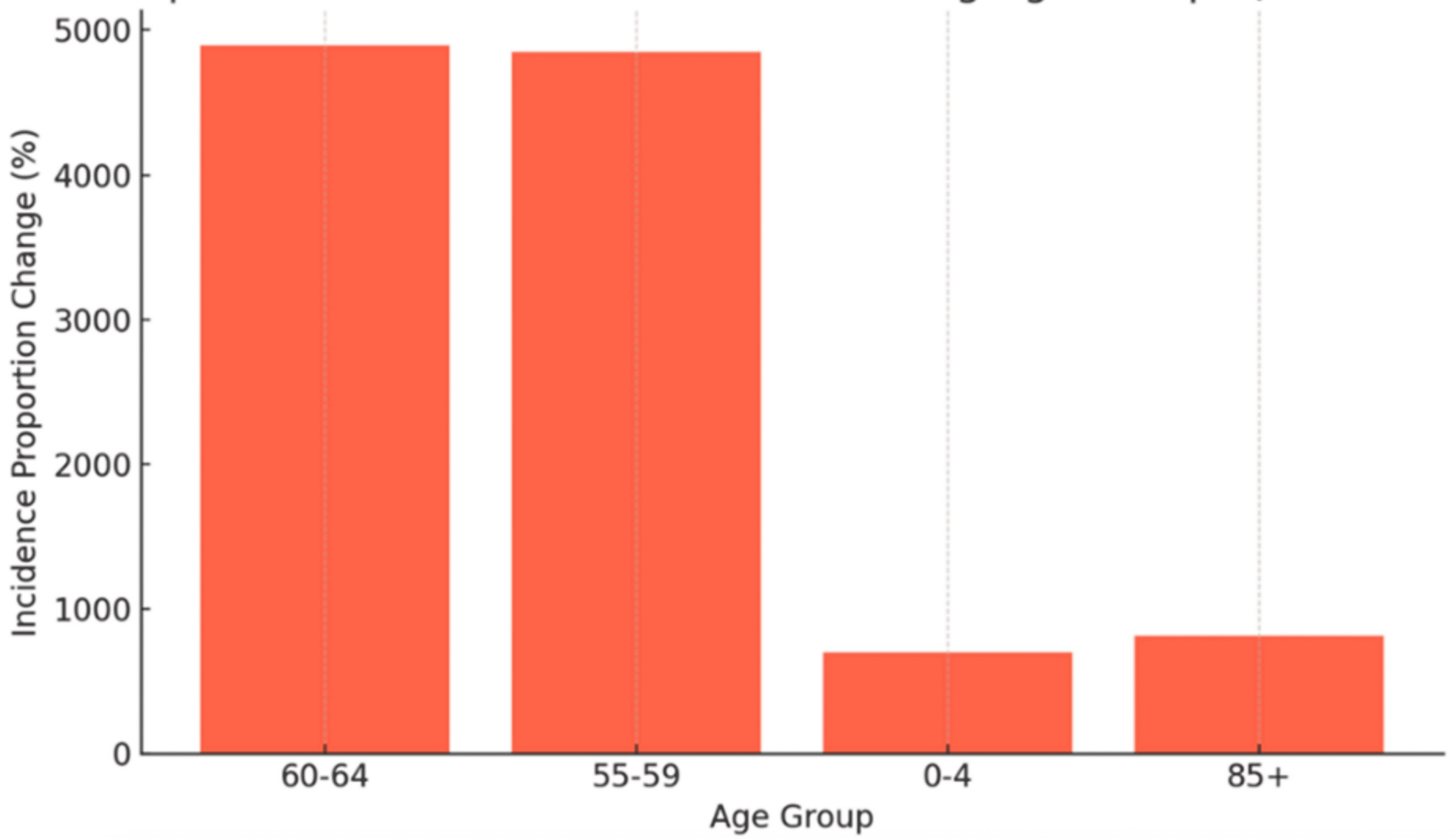
Comparison of Fastest and Slowest Growing Age Groups (2015-2025)

Across age groups, the incidence of MMA increased consistently. The youngest cohort (ages 0-4) experienced a 698% rise, and those aged 5-9 showed a 1,134% increase. Adolescents and young adults (ages 10-24) demonstrated particularly high increases, with a 1,717% increase in those aged 10-14 and a 2,844% rise in individuals aged 20-24. Adults aged 25-49 had increases exceeding 3,000%, peaking in the 45-49 age group at 4,032%. Older adults also experienced substantial growth: those aged 55-59 increased by 4,849%, 60-64 by 4,853%, and individuals aged 65-84 showed increases ranging from 4,600% to 5,300%. Even among those aged >85, the incidence increased by 817%. Figure 1 illustrates these changes in incidence across all age groups, emphasizing the steep escalation with advancing age, while Figure 2 compares the fastest- and slowest-growing age groups to underscore these disparities.

**Figure 3:**
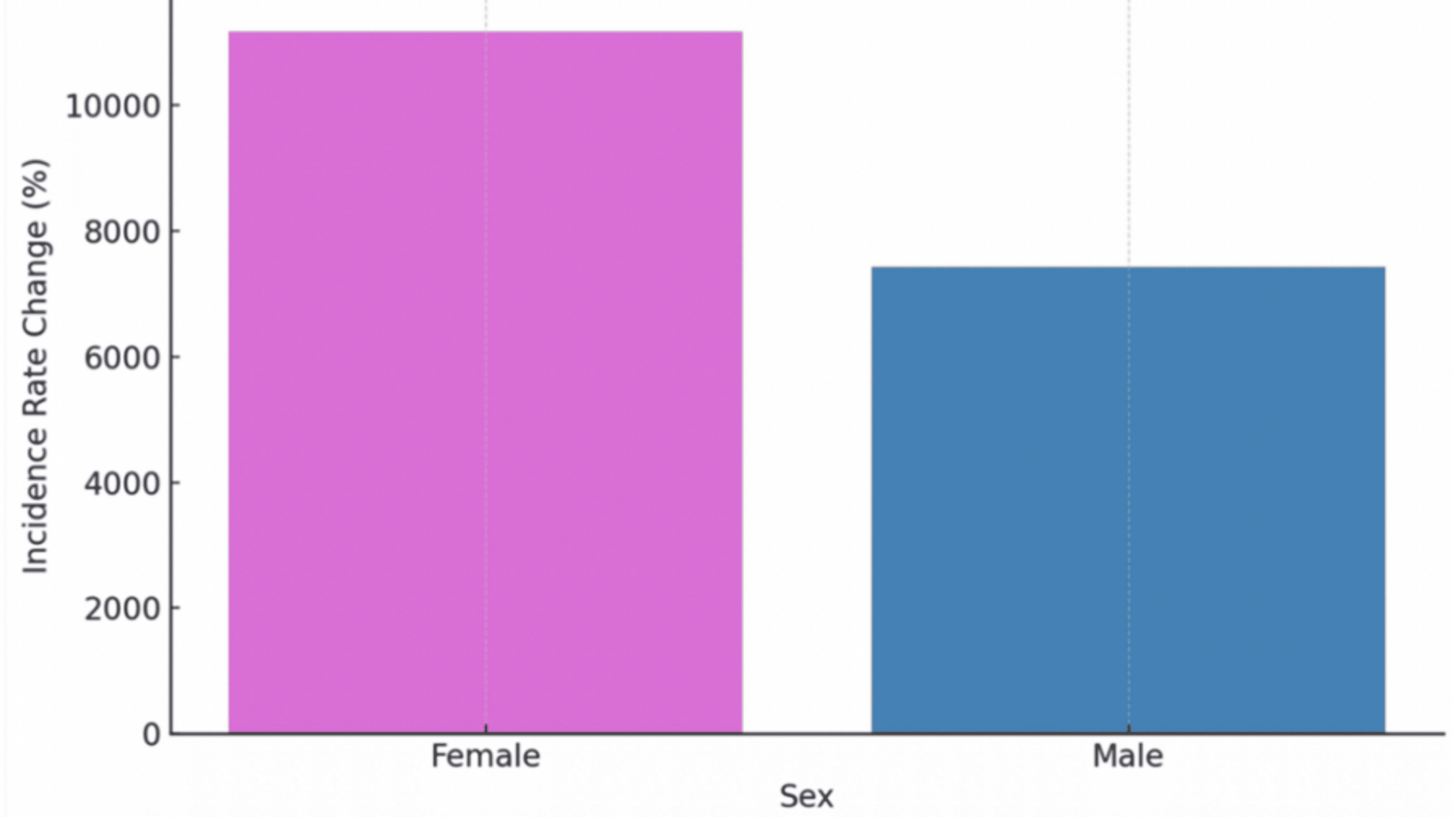
Change in Incidence Rate of Mammalian Meat Allergy by Sex

When stratified by sex, females experienced greater increases than males across all metrics: a 6,971% increase in incidence proportion, a 6,931% increase in prevalence, and an 11,169% increase in incidence rate. Males, by comparison, had a 4,620% increase in incidence proportion, a 4,616% increase in prevalence, and a 7,426% increase in incidence rate. These differences may reflect sex-based disparities in immune response, clinical awareness, or healthcare-seeking behavior. Figure 3 depicts the change in incidence rate of MMA by sex, highlighting the disproportionately sharper increase among females.

Analysis by race and ethnicity revealed consistent and substantial increases across all groups. Black or African American individuals experienced an increase from 400 new cases in 2015-2020 to 22,540 in 2021-2025, reflecting a 5,535% increase. American Indian or Alaska Native individuals saw cases rise from 1,006 to 56,620 (5,528%). White individuals rose from 268 to 15,106 cases (5,537%), and Asian individuals from 136 to 7,660 cases (5,532%). Native Hawaiian or Other Pacific Islander cases increased from 140 to 7,896 (5,540%), and the Other Race and Unknown Race categories rose by 5,541% and 5,550%, respectively. Hispanic or Latino individuals experienced a 5,523% increase (from 228 to 12,821 cases), while those identifying as Not Hispanic or Latino increased from 338 to 19,015 cases (5,526%). These detailed trends, including shifts in incidence proportion, prevalence, and incidence rate by race and ethnicity, are presented in Table 1, while the absolute counts of newly diagnosed cases with percent increases by group are summarized in Table 2.

**Table 1:** Race and Ethnicity - Stratified Allergy Trends

Group	Incidence Proportion 2015-2020	Prevalence 2015-2020	Incidence Rate 2015-2020	Incidence Proportion 2021-2025
American Indian or Alaska Native Individuals	0.0	0.0	0.0	0.00004936581
Asian Individuals	3.2234118 × 10^-6^	3.2234118 × 10^-6^	2.665872 × 10^-9^	6.678621 × 10^-5^
Black or African American Individuals	2.5155482 × 10^-6^	2.5155482 × 10^-6^	2.0179618 × 10^-9^	0.00019652076
Native Hawaiian or Other Pacific Islander Individuals	0.0	0.0	0.0	6.8849076 × 10^-5^
White Individuals	2.3790233 × 10^-6^	2.4125304 × 10^-6^	1.996179 × 10^-9^	0.0001317107
Hispanic or Latino Individuals	2.556732 × 10^-6^	2.556732 × 10^-6^	2.183871 × 10^-9^	0.000111783804
Not Hispanic or Latino Individuals	2.905293 × 10^-6^	2.9405237 × 10^-6^	2.2423063 × 10^-9^	0.00016578929

**Table 2:** Total Case Numbers by Race and Ethnicity

Group	2015-2020 Cases	2021-2025 Cases	Percent Increase
American Indian or Alaska Native Individuals	1,006	56,620	5,528%
Asian Individuals	136	7,660	5,532%
Black or African American Individuals	400	22,540	5,535%
Native Hawaiian or Other Pacific Islander Individuals	140	7,896	5,540%
White Individuals	268	15,106	5,537%
Other Race	90	5,077	5,541%
Unknown Race	36	2,034	5,550%
Hispanic or Latino Individuals	228	12,821	5,523%
Not Hispanic or Latino Individuals	338	19,015	5,526%

Age-stratified trends within racial and ethnic groups showed that incidence rose more sharply among older individuals. For example, among White individuals, the incidence proportion increased by 6,054% for those aged 40 and older, compared to 4,662% for those under 40. Similar disparities were observed in Black or African American individuals (9,530% for ≥40 vs. 5,117% for <40), and Hispanic or Latino individuals (7,678% vs. 4,483%). Table 3 details these age-related trends within each racial group, illustrating how the burden of MMA is disproportionately increasing among older adults across all demographics.

**Table 3:** Age Within Race Group Trends

Race	Age Group	Incidence Proportion (2015-2020)	Incidence Proportion (2021-2025)	Percent Increase
White Individuals	<40	2.1 × 10^-6^	0.0001	4,662%
White Individuals	40+	2.6 × 10^-6^	0.00016	6,054%
Black or African American Individuals	<40	2.3 × 10^-6^	0.00012	5,117%
Black or African American Individuals	40+	2.7 × 10^-6^	0.00026	9,530%
Hispanic or Latino Individuals	<40	2.4 × 10^-6^	0.00011	4,483%
Hispanic or Latino Individuals	40+	2.7 × 10^-6^	0.00021	7,678%

**Figure 4:**
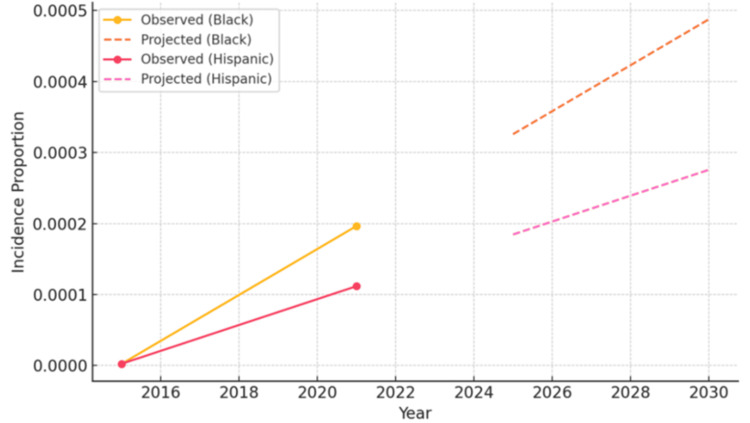
Projected Future Trends in Mammalian Meat Allergy Among Black and Hispanic Populations

Predictive modeling for 2025-2030 indicates continued upward trends. Among Black individuals, the incidence proportion is projected to rise from 0.00020 to 0.00031 (a 57% increase), and among Hispanic individuals, from 0.00011 to 0.00019 (a 72% increase). These forecasts suggest a continued and disproportionate burden among historically underserved populations. Figure 4 illustrates these projected increases in MMA incidence within Black and Hispanic populations through 2030.

## Discussion

The breakdown of MMA incidence by age within racial and ethnic groups demonstrates a consistent rise across all demographics, with particularly steep increases among adults aged 40 and older. While the condition remains rare overall - rising from an estimated 180 new diagnoses in 2015-2020 to 10,132 in 2021-2025 - these findings underscore its growing clinical recognition. In the White population, individuals under 40 experienced a 4,662% increase in incidence proportion between the two time periods, whereas those over 40 saw a 6,054% increase. Among Black individuals, the increase was 5,117% in the younger cohort, compared to 9,530% in those over 40. Hispanic individuals followed a similar pattern, with a 4,483% rise in those under 40 and a 7,678% increase in older adults. These trends suggest that although MMA is increasing across all age groups, the largest proportional growth is occurring in middle-aged and older populations. This may reflect a delayed onset pattern, heightened exposure risk in later life, or improved diagnostic recognition in older adults.

The emergence of such stark differences by age within each racial group suggests that MMA may be underrecognized or underdiagnosed in earlier decades of life, only becoming more apparent as individuals age. Delayed sensitization due to tick bites is a primary factor in AGS development, with reactions typically occurring two to six hours after meat consumption, complicating timely diagnosis. A systematic review reported a mean age of 51.3 years among AGS cases, supporting the notion of increased incidence in later adulthood [12]. The uniformity of this age-related trend across racial and ethnic groups strengthens the likelihood of a biological or environmental component driving increased incidence in later adulthood. Additionally, the University of North Carolina Allergy Clinic conducted a study that found 77% of AGS patients were aged 40 or older at symptom onset [13]. These findings, therefore, do not support the notion that awareness is needed in all age groups but, rather, contribute to the notion that increased medical observation and environmental exposures in older adults may lead to these increased age-related trends.

Several potential explanations may underlie this age-related disparity. One possibility is the increased likelihood of cumulative environmental exposures, such as tick bites that can trigger AGS, a leading cause of MMA. Older adults may also be more likely to seek medical evaluation for atypical symptoms or undergo diagnostic testing, resulting in greater detection rates. However, a review published in the Journal of Applied Gerontology found that older adults often avoid seeking medical help due to several factors, such as informal support, negative perceptions of healthcare providers, and perceived threats to independence [14]. Additionally, delayed-onset allergic reactions, which are increasingly recognized in adults, could account for the higher rates observed in individuals aged 40 and above. A study published in the Journal of Allergy and Clinical Immunology highlighted that half of U.S. adults with a food allergy report developing at least one of their allergies during adulthood [15]. These findings emphasize the importance of expanding allergy screening and education beyond pediatric populations, including adults and older individuals.

The predictive modeling supports these observations, forecasting a continued rise in incidence among high-risk groups through 2030. The incidence proportion in the Black population is projected to increase by an additional 57%, while the Hispanic population may see a 72% increase. These projections reflect not only ongoing risk but also the potential for widening disparities if targeted public health measures are not implemented. A study published in JAMA in 2023 found that food allergy prevalence was highest among Black, Hispanic, and Asian populations [16]. Without proactive interventions, the burden of MMA will likely intensify, particularly in communities already experiencing disproportionate increases. It is important to note that, while these findings demonstrate robust descriptive trends, our reliance on EHR data and lack of formal statistical tests limit definitive inferences, as discussed further below. Nonetheless, the consistent and substantial increases across demographic groups underscore an emerging clinical and public health concern warranting further investigation.

Despite the relative increases, the absolute number of cases remains low. Therefore, while surveillance is warranted, this should not be interpreted as a widespread epidemic. In light of these trends, there is a clear need for comprehensive, regionally focused public health strategies that address both prevention and early diagnosis. Clinicians should maintain a high index of suspicion for MMA in adult patients presenting with delayed allergic symptoms, especially in areas endemic to tick exposure. Public health messaging should be tailored to high-risk populations, emphasizing tick-bite prevention, allergy awareness, and prompt evaluation of symptoms. By focusing on older adults and communities of color, where the allergy is rising most rapidly, we can begin to mitigate the future burden and improve health equity in allergy diagnosis and management.

Nonetheless, this study has limitations. These include the potential influence of improved diagnostic coding practices and heightened clinical awareness over time, which may partially account for the observed increases, rather than reflecting solely a true rise in disease occurrence. There is also incomplete capture of asymptomatic or undiagnosed cases, and we were unable to directly measure tick exposure among affected individuals, limiting assessment of environmental contributions. While this study provides robust descriptive trends stratified by age, sex, race, and ethnicity, formal statistical comparisons of interaction effects or significance testing between demographic groups were beyond the scope of this EHR-based analysis. Future studies employing multivariable regression could better elucidate these relationships. Despite these constraints, the study’s use of a large, nationally representative, and racially and ethnically diverse cohort remains a major strength, enhancing generalizability. The observed patterns strongly support MMA as an emerging and increasingly recognized public health concern that warrants further research and targeted interventions.

## Conclusions

This study highlights a substantial rise in MMA diagnoses across all demographics, particularly among middle-aged adults and communities of color. Though still uncommon, this trend suggests an emerging health concern requiring clinician awareness, especially in regions endemic to tick exposure. These findings support the development of targeted guidelines and future research into preventative measures, including tick-bite avoidance strategies and diagnostic algorithms that incorporate delayed-onset food allergies.
